# TryTransDB: A web-based resource for transport proteins in Trypanosomatidae

**DOI:** 10.1038/s41598-018-22706-x

**Published:** 2018-03-12

**Authors:** Krushna Sonar, Ritika Kabra, Shailza Singh

**Affiliations:** National Centre for Cell Science, NCCS Complex, Ganeshkhind, SP Pune University Campus, Pune, 411007 India

## Abstract

TryTransDB is a web-based resource that stores transport protein data which can be retrieved using a standalone BLAST tool. We have attempted to create an integrated database that can be a one-stop shop for the researchers working with transport proteins of Trypanosomatidae family. TryTransDB (Trypanosomatidae Transport Protein Database) is a web based comprehensive resource that can fire a BLAST search against most of the transport protein sequences (protein and nucleotide) from Trypanosomatidae family organisms. This web resource further allows to compute a phylogenetic tree by performing multiple sequence alignment (MSA) using CLUSTALW suite embedded in it. Also, cross-linking to other databases helps in gathering more information for a certain transport protein in a single website.

## Introduction

Trypanosomatidae Transport Protein Database (TryTransDB), is a web based comprehensive resource that harbors protein and nucleotide sequences belonging to various superfamilies of transport proteins found in the members of the Trypanosomatidae family. The species of this family are parasitic protozoans in nature and have a single flagellum along with kinetoplastid which forms as an important distinguishing feature. The parasites can be dixenous or monoxenous depending on the number of hosts needed to complete their life cycle. The type of hosts ranges from insects which are usually the vectors to hosts like plants (eg. Phytomonas genera) and animals (eg. Trypanosoma and Leishmania genera)^1^. Organisms belonging to Leishmania^2^ cause Leishmaniasis while *Trypanosoma cruzi* and *Trypanosoma brucei* cause Chagas^3^ and sleeping sickness^4^ respectively. These diseases have serious deleterious effects or sometimes even fatal leishmaniasis^5^.

The above-mentioned diseases can be localized in many parts of India, Africa and recently in South America^6^. Numerous drugs are available to protect against the infection but all of these have their respective pros and cons^6,7^. Published research repertoire says that resistance has been observed against Trypanosomiasis^8^ and Leishmaniasis^9^. This resistance is usually attained by the virtue of transport proteins found on the cell membrane that is responsible for pumping out the drugs out of the cell^9,10^. One way to inhibit the function of these transport protein is to have certain types of molecule, be designed that will hinder their function either by inhibition or reducing their efficacy.

Studies have shown that these transport proteins like Major Facilitator Superfamily (MFS) and ATP-binding Cassette (ABC) protein can be used as potential drug targets^11,12^. MFS and ABC are Transport Proteins, that are responsible for the transportation of molecules into and outside of the cell. These proteins are located either on the cell membrane or buried inside it where they span the complete width of the membrane.

Six types of superfamilies of transport proteins that are found in Trypanosomatidae family. ATP binding Cassette transporters (ABC), protein is located on the extracellular region of membrane and transportation is done by the hydrolysis of an ATP molecule. Various models of mechanism have been proposed^13^. Major Facilitator Superfamily (MFS), is the most ubiquitous, present in many organisms. Originally assumed function was sugar uptake just to later find out there are more biomolecules that are transported. The “rocker-switch model” is the proposed mechanism of function^14^. Resistance-Nodulation-Division (RND), proteins belonging to this family actively transport. This protein is a part of a complex comprising two more proteins that together span the outer and the periplasmic membrane. The association of these three results in transport, usually the proteins in these family allow the bacteria to gain resistance to a drug^15^. Amino Acid-Polyamine-Organocation Family (APC), protein has 12 transmembranes and the mechanism is known as “Rocking-bundle”^16,17^.

Multidrug/Oligosaccharidyl-lipid/Polysaccharide (MOP) superfamily: The structure of these proteins comprises of twelve transmembrane helices and they act as cation antiport. Voltage-gated Ion Channel (VIC), they form ion channels and are specific to ions like sodium, potassium, calcium and chloride ions. They are uniport type transporters and have six transmembrane spanning segments.

Owing to the importance of these transport protein not only as drug target but also as a medium for the cell to be in contact with the environment, TryTransDB is made to cater the needs of researchers who are working in the respective fields. Since TryTransDB comprises of sequences only belonging to the Trypanosomatidae family, it becomes easy to filter out sequences of user’s interest using the standalone BLAST incorporated in it. CLUSTALW^18^, using its default values, helps in calculating the phylogeny between the BLAST hits. Crosslinking is provided in order to get a wider spectrum of information from databases like KEGG^19^, NCBI Pfam^20^, EMBL-EBI Pfam^20^ and NCBI Genome Browser^21^ for the respective hits.

Information regarding the organism family with its medicinal importance along with the lifecycle of Trypanosomiasis, Leishmaniasis and the transport protein family is also available which gives a small gist of what the database is all about. Post BLAST run, a user can also download sequences from the BLAST output using the “DOWNLOAD” option which can be further used for more resource intensive phylogeny algorithms or for MEME suite which is cross-linked in the navigation bar. Users with novel sequences can even submit their sequences using the SeqSub tool in TryTransDB.

## Materials and Methods

The front end of TryTransDB is developed using HTML. Apache was used to host the website and back end handling was done using PHP5 and PERL. Two databases were made, one for BLAST standalone software while one for phylogeny and downloading of sequences was made using MySQL.

### Data collection and compilation

**Figure 1:**
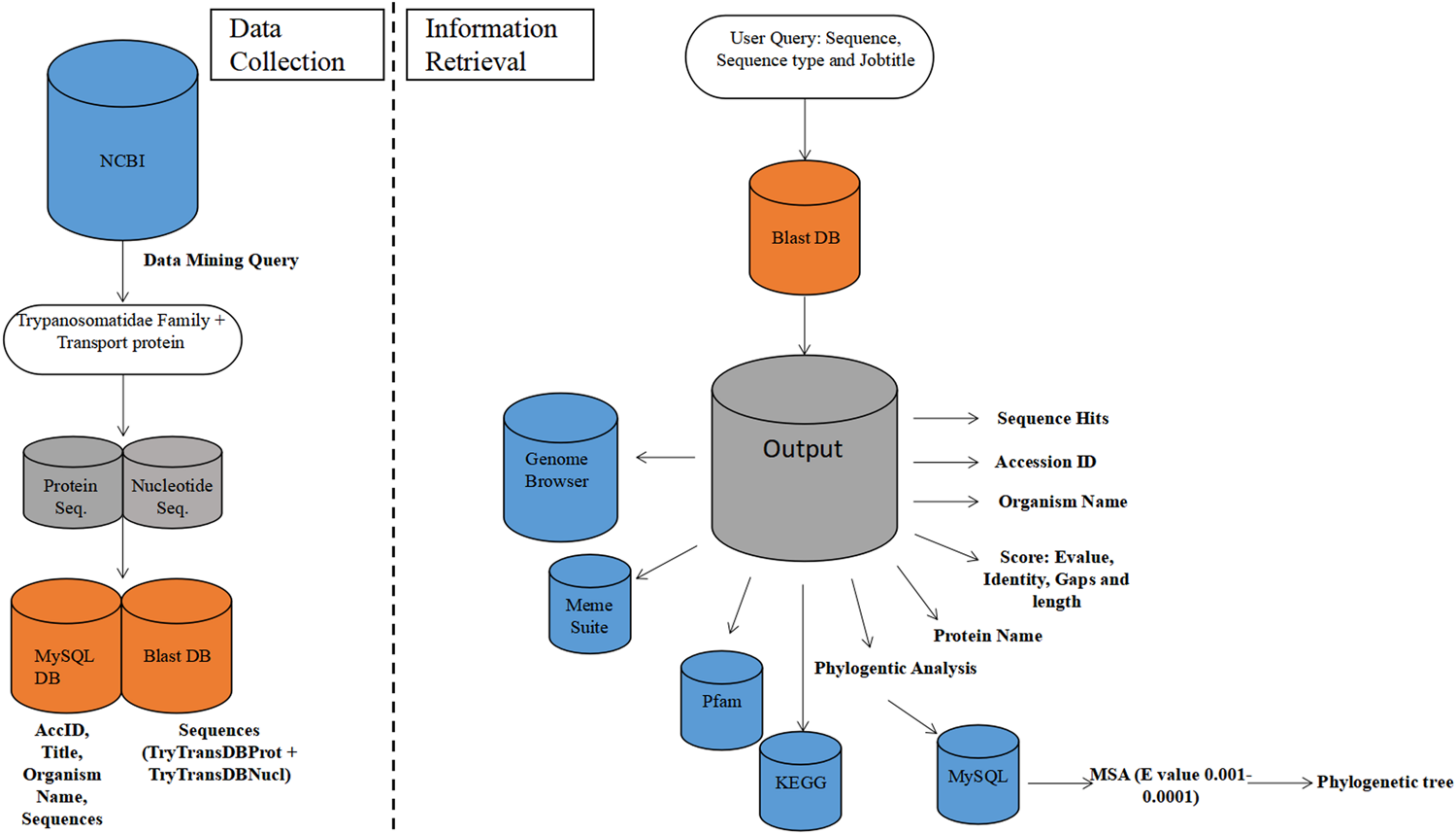
TryTransDB architecture.

Trypanosomatidae family members were selected from the taxonomy database of NCBI. Transport proteins; ATP-binding Cassette (ABC) Superfamily, Major Facilitator Superfamily (MFS), Resistance-Nodulation-Cell Division (RND) Superfamily, Amino Acid - Polyamine - Organocation (APC) Family, Multidrug/Oligosaccharidyl - lipid/Polysaccharide (MOP) Flippase Superfamily, Voltage-gated Ion Channel (VIC) Superfamily were selected. An appropriate query was fired onto NCBI (https://www.ncbi.nlm.nih.gov/) using an in house script to download the protein and nucleotide. Around 754 protein sequences and 254 nucleotide sequences were retrieved and stored in TryTransDB as shown in Fig. 1.

### Data Availability

The data present in the database can be accessed on the website http://43.227.133.106/homePage.php.

## Result

The home page gives an introduction to TryTransDB, wherein, on the right side is the navigation bar.

### Usage of TryTransDB

**Figure 2:**
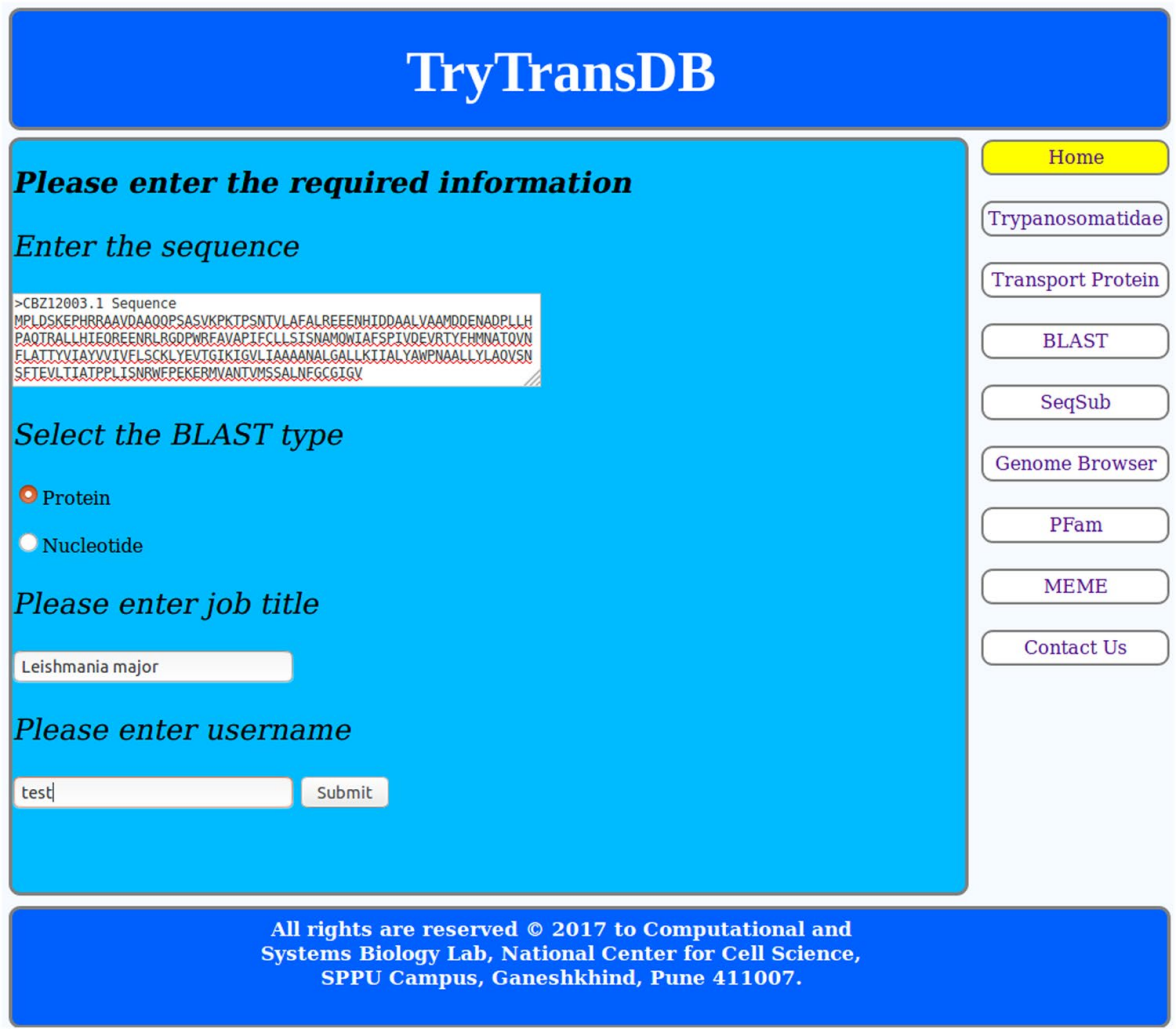
BLAST page. Details sequence, BLAST type, job title and username are to be filled in the given space.

To visualize the working of TryTransDB, let’s consider a case where the user wants to know if there are any organism and transport protein available for the MFS transport protein, user has sequenced. The input is protein sequence as shown in Fig. 2 and is taken from the existing entry from NCBI (CBZ12003.1).

### BLAST output and KEGG

**Figure 3:**
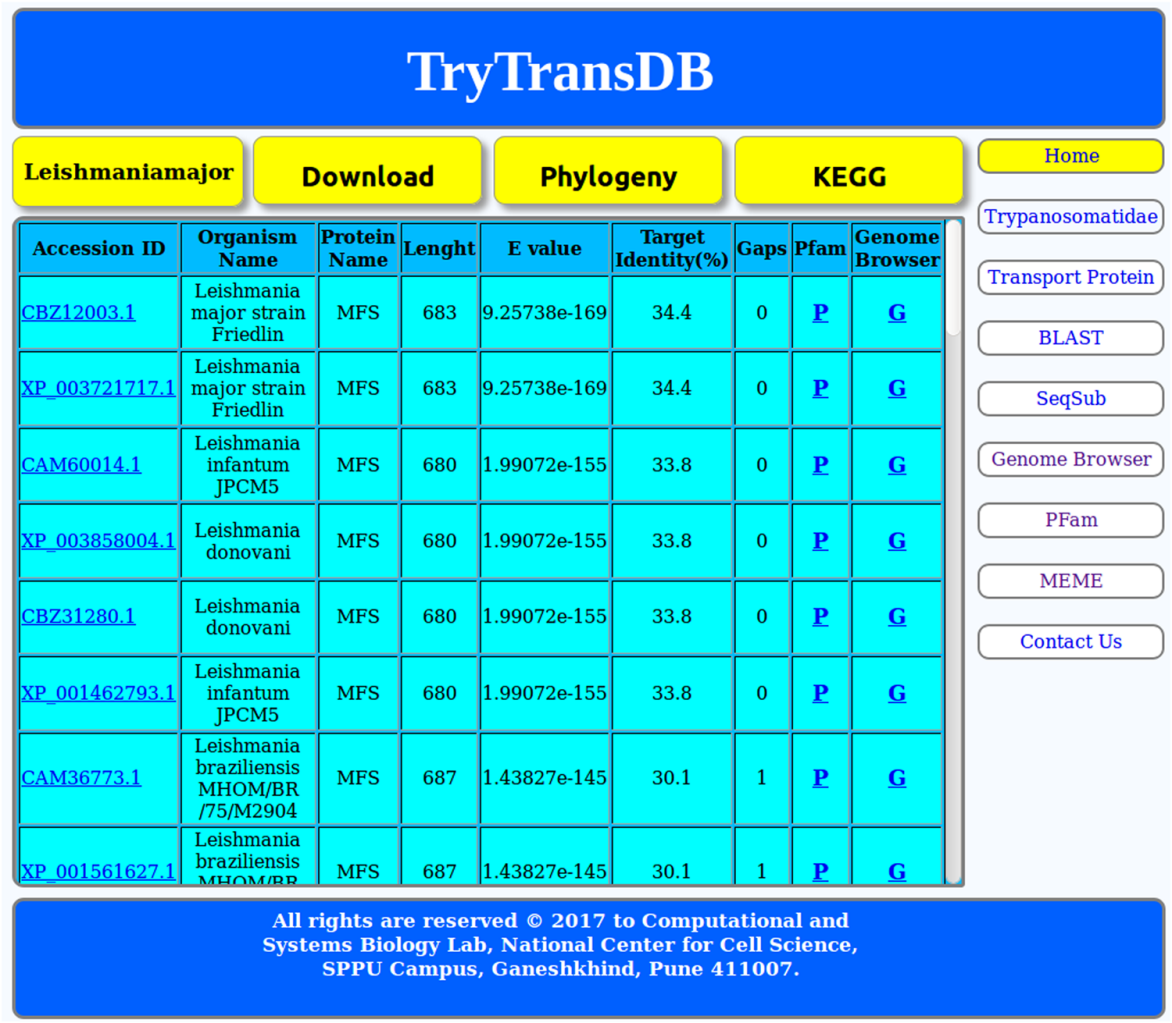
Output page.

After entering the required fields in the “BLAST” page. The standalone BLAST using its local search algorithm checks sequences for similarity and identity against the query sequences and displays information as represented in Fig. 3.

BLAST output. Information like the hits along with Accession ID (linked to NCBI), Organism name, Protein name, Lenght, E value, Target Identity(%), Gaps and Pfam, Genome Browser (linked to NCBI) and KEGG are mentioned. The first hit confirms the BLAST has correctly identified the organism.

**Figure 4:**
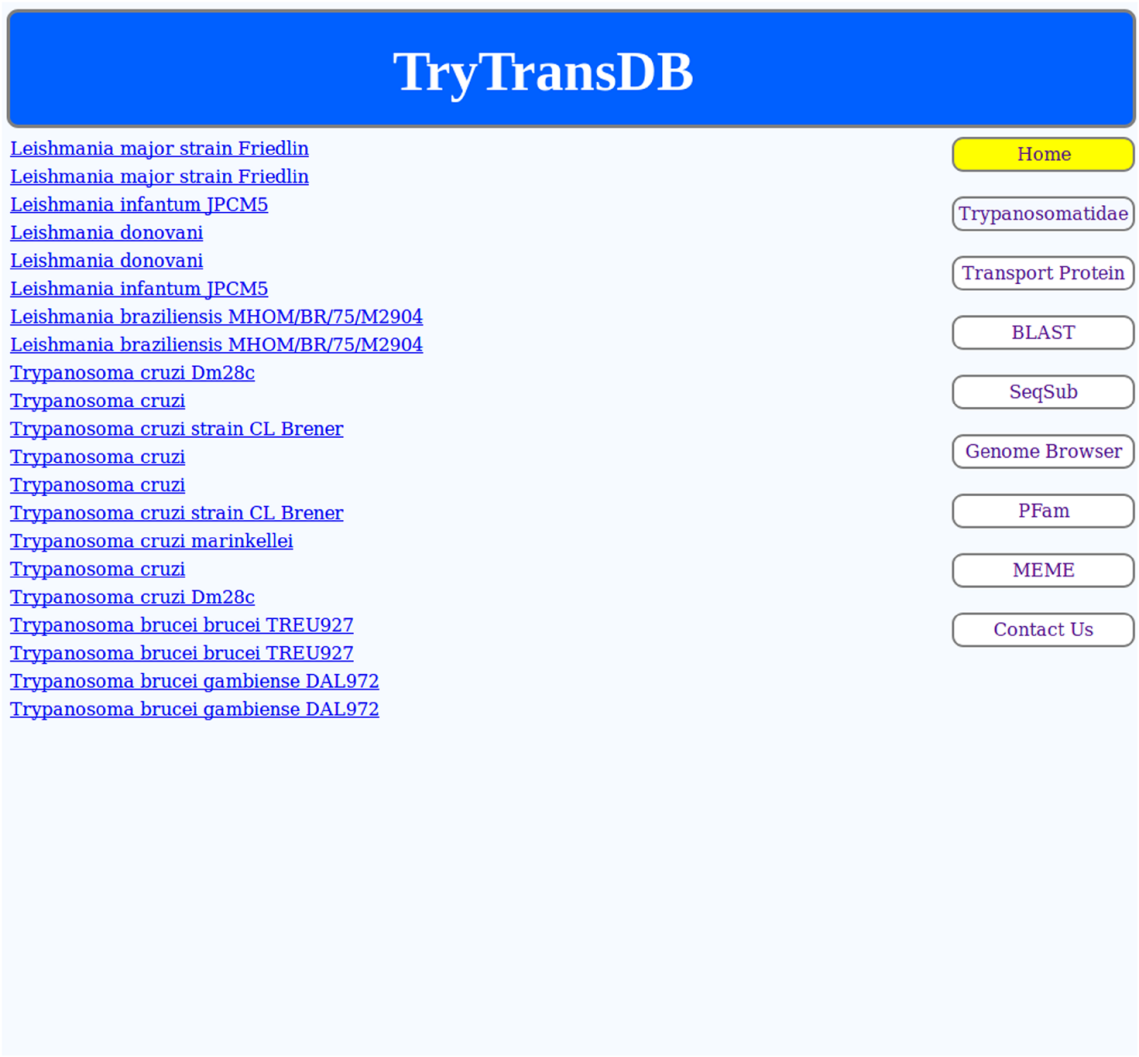
KEGG output. Hyperlinks to the KEGG database are displayed.

KEGG links can be checked by clicking on the “KEGG” hyperlink (Fig. 4). A new tab is opened which comprises of only those entries for organisms that are present in KEGG’s database. So, it is likely that some organisms and their strains that have come as a hit will not appear in the KEGG list.

### Phylogeny

**Figure 5:**
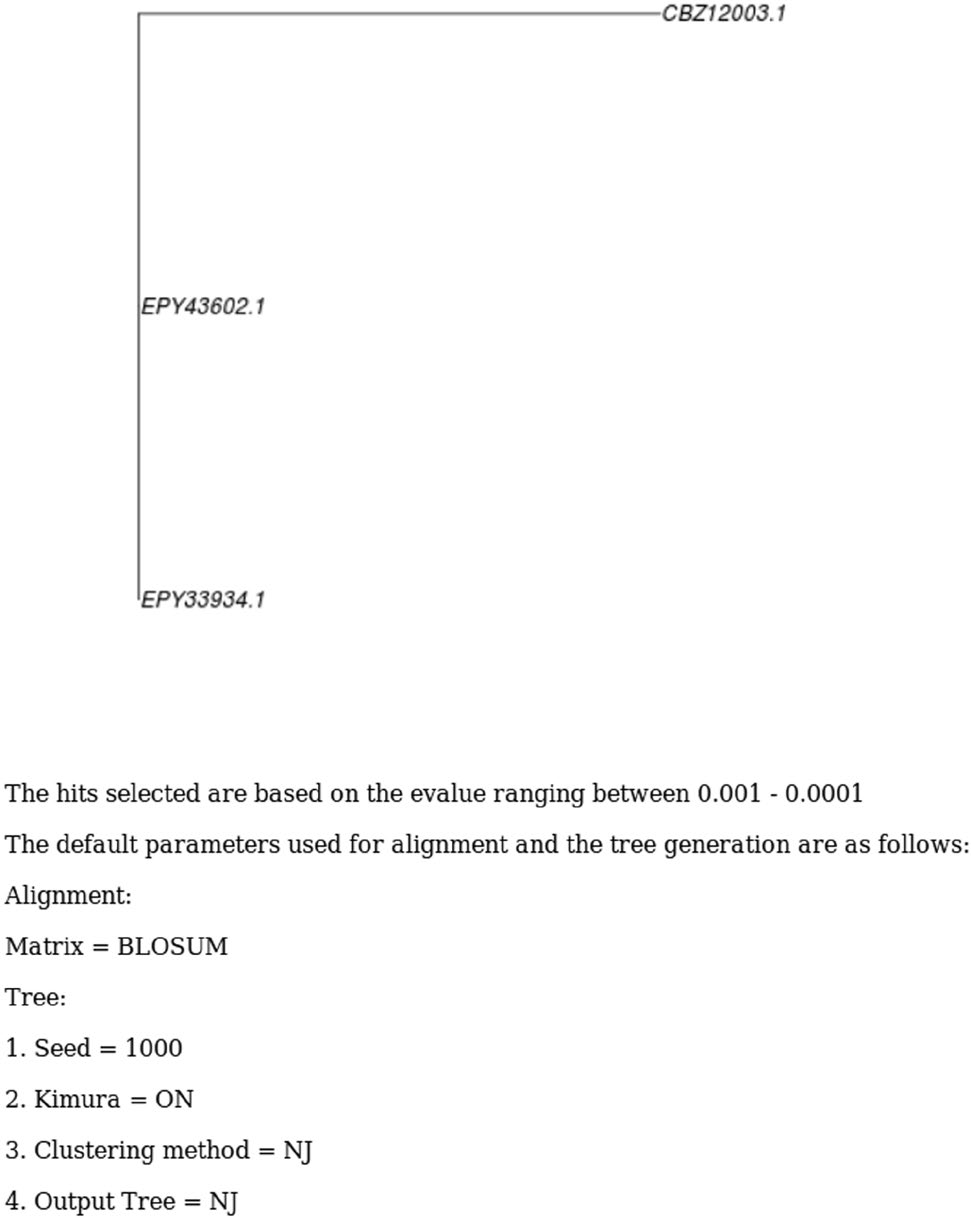
Phylogeny output. Newick form phylogenetic tree generated using CLUSTALW.

A phylogenetic tree can also be generated by clicking on the “Phylogeny” link. TryTransDB computes the phylogenetic by invoking ClustalW and runs by using some default values. The database comprises of protein sequences whose E value is very close to each other and hence for the ease of crowding an E value cutoff (0.001–0.0001) has been applied for protein phylogenetic tree generation. This is not applied while calculating nucleotide phylogenetic tree generation since the number of sequences submitted are very less. Trees generated are represented in Newick format with the default conditions used and mentioned below (Fig. 5).

### Pfam

**Figure 6:**
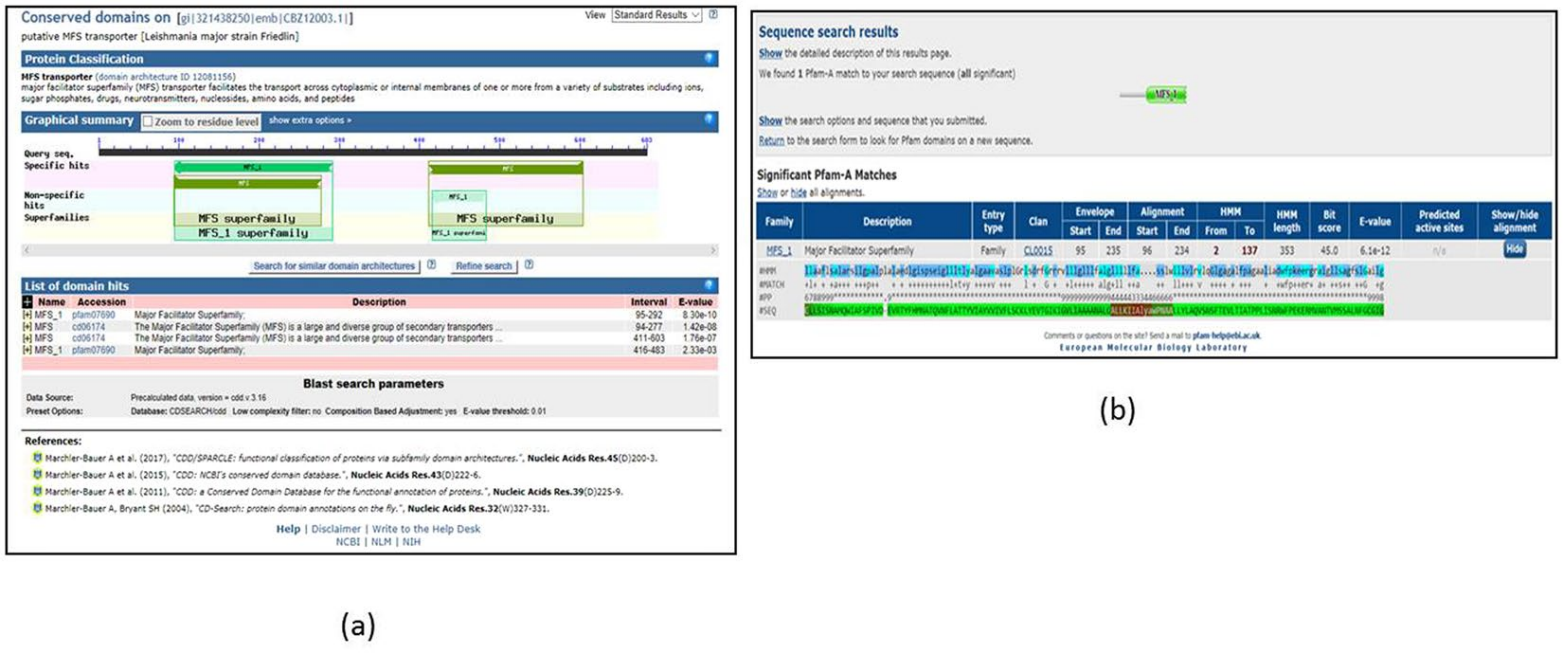
(**a**) Pfam output from NCBI. (**b**) Pfam output from EMBL database.

From TryTransDB, Protein family, information can be accessed for the sequence in the output in this database. Pfam information is taken from NCBI and EMBL. In the output page, Pfam NCBI data can be accessed by clicking on “P” symbol (Fig. 6a) present in every row. To access the Pfam information from the EMBL database, there is Pfam button in the right-hand panel. This will redirect the user to the EMBL-EBI Pfam webpage. Of many types of input the database can take, one is sequence which can be downloaded using the “Download” in the top panel of the output page (Fig. 6b).

### Genome Browser

**Figure 7:**
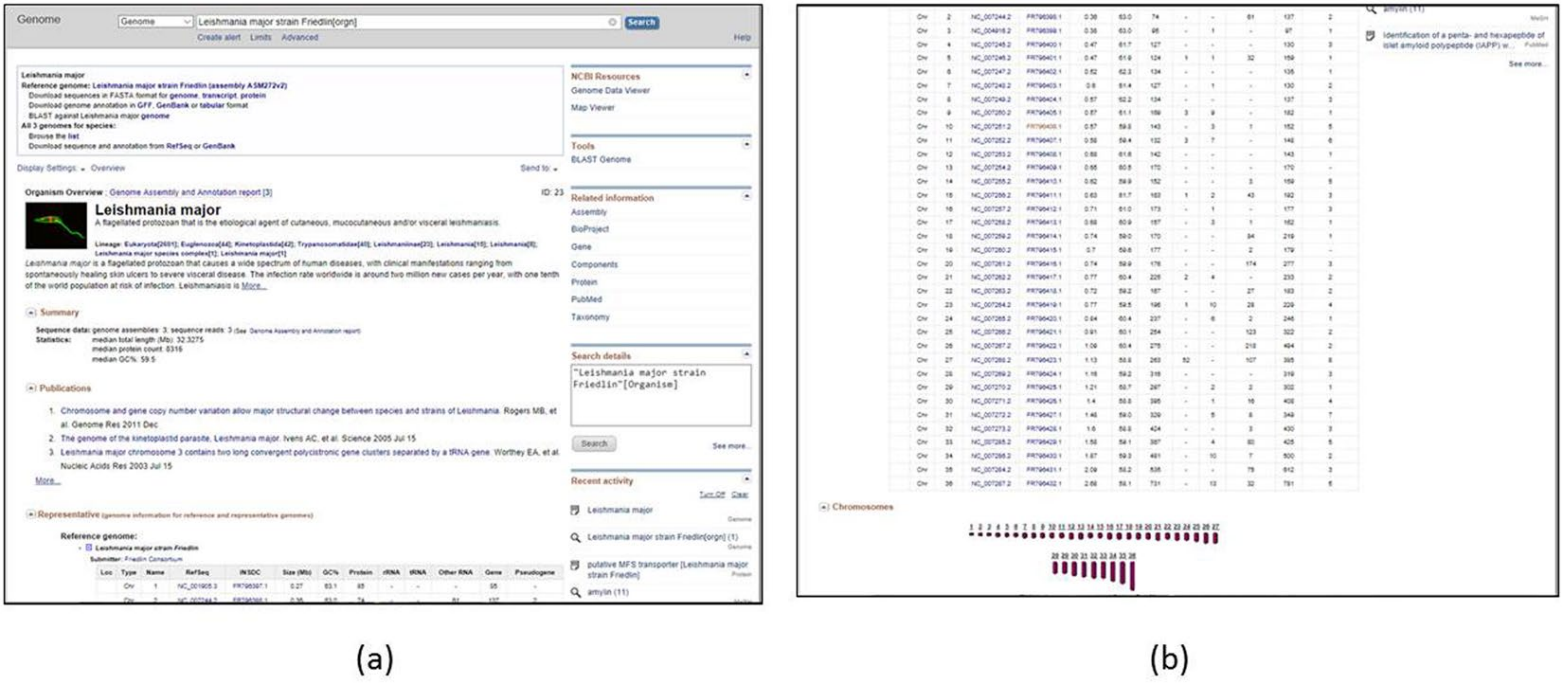
(**a**),(**b**) Genome output from NCBI for the selected entry.

Information like the location of the protein gene in the chromosome and additional details of the chromosome can be accessed through “Genome Browser”. By clicking on “G” symbol in the output page on every row of the result. This will redirect to the genome information of the respective entry in the NCBI (Figs. 7a,b).

### Sequence submission

If the user wishes to submit a novel sequence, it can be done via the “SeqSub tool”. Sequence and the job title should be entered in the respective fields and a BLAST search is fired. If the sequence already exists in the database, it will prompt about the same, whereas, if the sequence is not present, the sequence will be manually curated and later added to the database, in a period of three weeks.

## Discussion

Transport proteins are the potential drug target site since their function can be hindered by the use of some small molecule which leads to no exchange of molecules or important environmental signals or the administered drug like miltefosine in terms of leishmaniasis can function and not thrown out. Using this sequence repertoire, one can start with post-sequencing processes like structure prediction and molecular dynamics simulation techniques to understand the functioning and mechanism of these proteins in great details along with additional information available from cross-linked databases. The sites content would be kept updating for greater contributions towards the significant functionality of the web resource.
